# A Very Early Diagnosis of Complete Androgen Insensitivity Syndrome Due to a Novel Variant in the AR Gene: A Neonatal Case Study

**DOI:** 10.3390/biomedicines12081742

**Published:** 2024-08-02

**Authors:** Rossella Ferrante, Stefano Tumini, Maria Alessandra Saltarelli, Sara Di Rado, Vincenzo Scorrano, Maria Lucia Tommolini, Mirco Zucchelli, Federico Lauriola, Gabriele Lisi, Giuseppe Lauriti, Nino Marino, Liborio Stuppia, Claudia Rossi, Ines Bucci

**Affiliations:** 1 Center for Advanced Studies and Technology (CAST), “G. d’Annunzio” University of Chieti-Pescara, 66100 Chieti, Italy; rossella.ferrante@unich.it (R.F.); sara.dirado@phd.unich.it (S.D.R.); vincenzo.scorrano@unich.it (V.S.); maria.tommolini@unich.it (M.L.T.); m.zucchelli@unich.it (M.Z.); liborio.stuppia@unich.it (L.S.); ines.bucci@unich.it (I.B.); 2 Department of Maternal and Child Health, UOSD Regional Center of Pediatric Diabetology, Chieti Hospital, 66100 Chieti, Italy; stefano.tumini@asl2abruzzo.it; 3 Department of Pediatrics, University of Chieti, 66100 Chieti, Italy; mariaalessandra.saltarelli@gmail.com (M.A.S.); federicolauriola@hotmail.it (F.L.); 4 Department of Innovative Technologies in Medicine and Dentistry, “G. d’Annunzio” University of Chieti-Pescara, 66100 Chieti, Italy; 5 Pediatric Surgery Unit, Maternal and Child Health Department, Pescara Public Hospital, 65121 Pescara, Italy; gabriele.lisi@unich.it (G.L.); giuseppe.lauriti@unich.it (G.L.); nino.marino@asl.pe.it (N.M.); 6 Department of Medicine and Aging Science, “G. d’Annunzio” University of Chieti-Pescara, 66100 Chieti, Italy; 7 Department of Psychological, Health and Territory Sciences, “G. d’Annunzio” University of Chieti-Pescara, 66100 Chieti, Italy

**Keywords:** disorders of sexual development, steroid profiling, next-generation sequencing, androgen receptor, androgen insensitivity syndrome

## Abstract

Androgen insensitivity syndrome (AIS) is one of the most common Disorders of Sexual Differentiation (DSDs). AIS is characterized by an X-linked recessive inheritance pattern associated with variants in the androgen receptor (AR) gene that affects the masculinization process in individuals with XY karyotype. Here, we report a neonatal case of a very early diagnosis of complete AIS due to a novel variant in the AR gene. In the present case, after the clinical evaluation, the infant has undergone the following tests: biochemical analyses, including newborn screening workflow, karyotype analysis, and Next-Generation Sequencing (NGS) panel of 50 genes involved in DSDs. The NGS analysis identified a missense variant, c.2108C>A, in the AR gene. According to a cytogenetic analysis, the patient presented a 46, XY karyotype, thus the resulting hemizygote for the AR gene variant. The variant is not currently described in the literature nor in the ClinVar database. However, according to computational models, the variant could have a pathogenetic effect. This clinical case reveals a novel variant of the AR gene with a possible pathogenetic effect associated with AIS and highlights the importance of a multidisciplinary approach for the timely diagnosis and appropriate follow-up of the patient.

## 1. Introduction

Androgen insensitivity syndrome (AIS) is one of the most common disorders of sexual development (DSD). AIS is a genetic X-linked condition due to abnormalities in the androgen receptor (AR) gene resulting in a reduction in the masculinizing action of testosterone in chromosomally male subjects [1]. The estimated prevalence is of 2:100.000 to 5:100.000 [2]. The pathogenesis of AIS is characterized by more than 1000 variants of (AR) coding genes that impair the receptor assembly leading to a variable insensitivity of the cells to the hormones [2]. Different types of mutations in the AR gene cause AIS: small insertions or deletions, complete and partial deletions of the gene, and point mutations. The vast number of different mutations in the AR gene is responsible for the clinical heterogeneity of the syndrome, which also depends on the quantity and quality of the residual receptors. However, there are four different types of mutations in this gene: point mutations, which can lead to the substitution of a single amino acid or the placement of a premature stop codon; frameshift mutations, caused by nucleotide insertions or the deletions of nucleotides; complete or partial gene deletions, usually complete or partial gene deletions, in most cases causing a complete protein deficiency; and intronic mutations in splice donor or acceptor sites. The update of new mutations is reported in the Clinvar database (https://www.ncbi.nlm.nih.gov/clinvar/?term=AR%5Bgene%5D&redir=gene accessed on 31 May 2023), in the AR gene mutation database, available at http://androgendb.mcgill.ca (accessed on 31 May 2023) and a detailed mutation map is also available at http://androgendb.mcgill.ca/map.gif (accessed on 31 May 2023) [2]. Therefore, a spectrum of phenotypes is observed. When the androgen insensitivity is complete (complete androgen insensitivity syndrome–CAIS), there is a fully developed feminine phenotype with normal external genitalia with a blind-bottom vagina of variable length. The internal genitalia are absent, and the testes are in the abdomen, in the inguinal canal, or *labia maiora* and may result in an inguinal hernia, which is the main clinical sign in a pre-pubescent girl [1]. Primary amenorrhea with normal breast development and sparse axillary and pubic hair are the diagnostic clues of CAIS at puberty. In the partial androgen insensitivity form (PAIS), different clinical phenotypes are reported. The newborns appear with incompletely developed male or female external genitalia (partially fused labio-scrotal folds or bifid scrotum, clitoral hypertrophy or micropenis, perineo-scrotal hypospadias, uro-genital sinus with blind vagina, palpable testes in an inguinal hernia, or labio-scrotal folds). Isolated clitoral hypertrophy and isolated micropenis can be observed in the severe and mild forms, respectively. There is also a mild androgen insensitivity syndrome form (MAIS), characterized by a normal male habitus with spermatogenesis defect or reduced secondary terminal hair, [2,3]. We describe a very early diagnosis of CAIS, in a 3-day-old 46 XY infant, due to a previously unreported variant in the AR gene. This case aims not only to describe a new variant of the AR gene, but also to highlight the importance of a multidisciplinary approach in allowing an early diagnosis of AIS.

## 2. Case Presentation

### 2.1. Case Report

A female newborn was referred to the Regional Center of Pediatric Diabetology and Endocrinology of Chieti for expert consultation about abnormal external genitalia.

The family history was unremarkable; the patient was firstborn, and her parents were not consanguineous. She was born at full term after a normal pregnancy; the neonatal period was physiological. On physical examination at birth, she was 51 cm tall and weighed 3.550 g; At three days of age, she was admitted to the Neonatal Department for the detection of gonadal formations in the *labia maiora* in the absence of any other symptom. 

The specific details of the physical examination and clinical evaluation are reported below.

### 2.2. Physical and Clinical Evaluation

On clinical evaluation, the genitalia showed normal *labia minora*, clitoris, and urethral meatus, and the vaginal opening was normally located, and testicles were palpable bilaterally in the hypertrophic *labia maiora*. A transabdominal ultrasound performed on the fourth day of life revealed the absence of the uterus and ovaries and the presence of bilateral testes in the *labia maiora*. 

## 3. Biochemical Studies

### 3.1. First- and Second-Level Testing by Newborn Screening Workflow

The newborn underwent newborn screening (NBS) for inherited metabolic disorders as required by national law [4,5]. The regional panel also includes screening tests for congenital adrenal hyperplasia other than for immunodeficiencies [6], for lysosomal storage diseases [7], and for spinal muscular atrophy (SMA) [8]. According to the laboratory protocol, dried blood spot (DBS) samples for NBS were collected at 48–72 h after birth by heel-pricking blood specimen onto an Ahlstrom 226 filter paper provided by PerkinElmer. Genital ambiguity was advised on the collection card. The details for quantitative determination in the DBS specimens of (17-OHP) by immunofluorimetric assays and of steroid profiling by ultra-performance liquid chromatography/tandem mass spectrometric (UPLC/MS/MS) are fully described in Supplementary Materials and specifically reported in Tables S1–S4.

### 3.2. DBS 17-OHP Determination and Steroid Profiling of the Neonatal Patient

**Figure 1:**
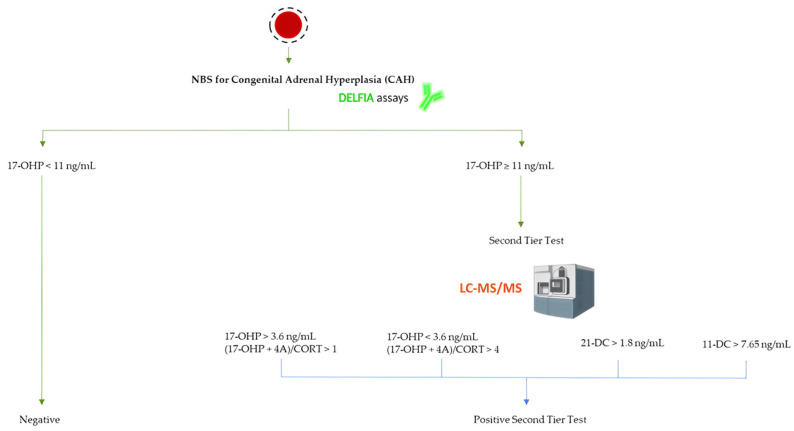
Screening algorithm for CAH in our NBS laboratory. The 17-OHP cut-off for term newborns is reported at the first-level test. If one of the criteria is met at the second-tier test, a positive result is obtained, and the newborn is recalled for further sample collection or for diagnostic confirmation. Image created with BioRender.com.

Figure 1 shows the workflow of the first- and second-tier testing strategy in NBS for CAH.

The NBS test by immunofluorimetric assay on the first DBS sample (49 h of life) revealed a normal level for the primary marker of CAH: 17-OHP = 1.0 ng/mL serum (n.v. < 11 ng/mL serum). More precisely, the 17-OHP cut-off levels at the NBS test were adjusted according to gestational age [9]. Even if the 17-OHP resulted normal, it was decided to perform the UPLC-MS/MS analysis for steroid profile as second-tier test that showed 17-OHP = 0 ng/mL (n.v. < 3.6 ng/mL), 21-deoxycortisol (21-DC) = 0 ng/mL (n.v. < 1.8 ng/mL), 11-deoxycortisol (11-DC) = 0.1 ng/mL (n.v. < 7.65 ng/mL), and the ratio between the sum of 17-OHP plus A4 concentration levels divided by the cortisol ones = 1 (n.v. < 1). Moreover, further analyses at 339 h of life confirmed normal level for 17-OHP at the NBS test, 17-OHP = 2.5 ng/mL serum (n.v. < 11 ng/mL serum), with the following steroid profiling at second-tier test: 17-OHP = 0.27 ng/mL (n.v. < 3.6 ng/mL), 21-DC = 0.11 ng/mL (n.v. < 1.8 ng/mL), 11-DC = 0.25 ng/mL (n.v. < 7.65 ng/mL), and the ratio 0.04 (n.v. < 1). 

### 3.3. Laboratory Results

The first-level laboratory tests revealed normal electrolyte, renal, liver, glucose, and thyroid profiles. The blood levels of serum anti-mullerian hormone (AMH), testosterone (T), and dihydrotestosterone (DHT) were elevated, while the glucocorticoid and mineralcorticoid profiles were within the normal range, as well as the 17α-Hydroxy-Progesterone (17-OHP) level (Table 1).

## 4. Molecular Studies

### 4.1. Chromosome Analysis

Chromosomal karyotyping by G-banding on metaphase chromosomes from peripheral blood lymphocytes manifested a 46, XY karyotype.

### 4.2. Genomic DNA Extraction

Genomic DNA was extracted from peripheral blood using the Blood DNA Extraction Kit (Zinexts Life Science Corp., Taipei, Taiwan) and MagPurix 12 EVO (Zinexts Life Science Corp., Taipei, Taiwan) according to the manufacturer’s protocol.

### 4.3. Genomic DNA Quantification

Genomic DNA was quantified using a Qubit 4 Fluorometer (Thermo Fisher Scientific, Waltham, MA, USA) according to the manufacturer’s protocol.

### 4.4. Next-Generation Sequencing (NGS)

The NGS analysis was performed with a custom Thermo Fisher NGS panel counting 50 genes involved in DSD (Table 2). Sequencing was carried out using Ion S5™ (Thermo Fisher Scientific, Waltham, MA, USA) and the NGS data analysis was executed via Ion Reporter Software 5.12 (Thermo Fisher Scientific, Waltham, MA, USA). 

The base coverage was over 20× in all the target regions, and the uniformity of base coverage was 98% in all the batches.

### 4.5. Confirmation of Gene Variant 

The NGS-detected variant was confirmed by Sanger sequencing through custom primers. The DNA sample was amplified via a polymerase chain reaction (PCR) performed in 30 μL reaction volume, including 22.25 μL of H_2_O, 3 μL of 10X PCR buffer, 2.1 μL of MgCl_2_ solution 25 mM, 0.5 μL of dNTPs 10 mM, 0.15 μL of AmpliTaq Gold polymerase, 1 μL of DNA, and 0.5 μL of forward and 0.5 μL of reverse primers. The amplification was carried out via a SimpliAmpTM thermal cycler (Termo Fisher Scientific, Waltham, MA, USA). The purification of the PCR products was performed via FastGene Gel/PCR Extraction (Nippon Genetics Europe, Düren, Germany), and PCR products have undergone Sanger sequencing via the SeqstudioGenetic Analyzer (ThermoFisher, Applied Biosystem, Foster City, CA, USA). 

### 4.6. Genetic Variant Description

**Figure 2:**
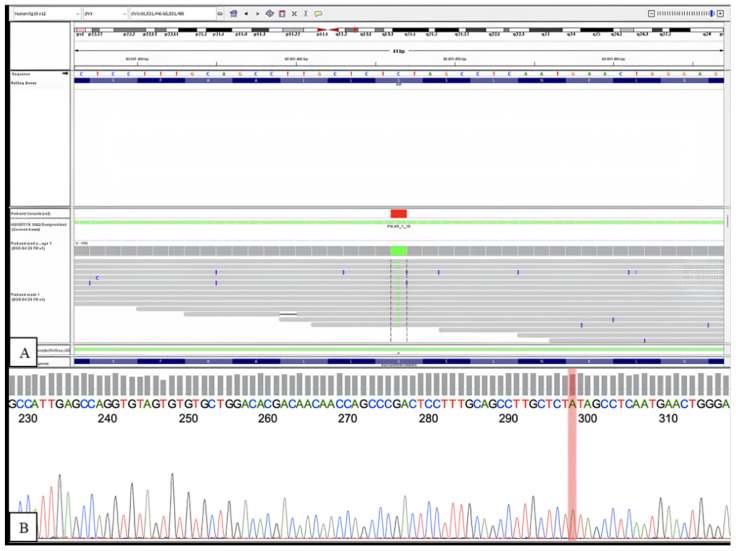
Visualization of the identified variant, c.2108C>A in the AR gene, on IGV (**A**) and in the sequence obtained by Sanger sequencing (**B**).

In this study, a c.2108C>A (p.Ser703Tyr) mutation was detected in the AR gene by the NGS analysis (Figure 2). The AR gene is located on chromosome Xq12 and encodes the androgen receptor, also known as the dihydrotestosterone receptor, involved in the development and maintenance of male sexual differentiation. The AR protein is an activated class I steroid receptor belonging to the nuclear receptor class. The identified missense variant in AR, c.2108C>A, causes the nucleotide substitution of the cytosine with an adenine at position 2108 onto exon 4, leading to the aminoacidic substitution of a serine with a tyrosine at codon 703 onto codified protein (p.Ser703Tyr). AR presents an X-linked recessive inheritance model. According to the cytogenetic investigation, the patient shows a normal 46, XY karyotype, thus resulting in the hemizygous state for the AR gene. AR c.2108C>A variant has not been described in the literature and ClinVar previously. According to the in silico predictors reported in the Varsome database, AR c.2108C>A variant results in a likely pathogenetic effect. 

## 5. Diagnosis and Follow-Up of the Patient

By analyzing the above clinical data, the preliminary diagnosis of CAIS was confirmed. 

The patient performed regular endocrinological visits that documented normal growth parameters. At the surgical evaluation, the transperineal ultrasonography documented the presence of a left gonad, with typical testicular ecostructure, at the inguinal canal/left *labia maiora*, and the endoabdominal ascent of the right gonad, also with typical testicular ecostructure, about 15 mm from the inner inguinal ring. Moreover, a short blind-ended vagina was described, while uterus/ovary was not observed. There was no obvious abnormality in the other organs.

## 6. Discussion

AIS is caused by genetic mutations in the AR gene resulting in varying degrees of atypical sex differentiation of 46, XY fetuses. Depending on the residual activity of the AR, the spectrum of clinical presentation ranges from normal phenotypic females to under-virilized and infertile phenotypic males with gynecomastia. Primary amenorrhea with normal breast development but sparse axillary and pubic hair is the main presentation in pubescent girls. In newborns, various genital anomalies are observed ranging from feminine phenotype with a blind-bottom vagina and with testes located in the abdomen, in the inguinal canal, or *labia maiora*, to isolated clitoral hypertrophy, or micropenis [1]. We describe a 3-day-old infant presenting with a bilateral palpable mass in the *labia maiora*. Laboratory, imaging, and molecular investigations were performed in order to confirm or exclude the clinical suspect of AIS. In pubertal girls, the differential diagnosis of CAIS includes all the causes of primary amenorrhea such as complete gonadal dysgenesis, Mayer/Rokitansky/Kuster/Hauser syndrome, and androgen biosynthesis disorders. Inguinal hernia is the typical presentation in infants but ambiguous genitalia can also be observed at birth in CAIS and in the most severe form of PAIS. The differential diagnosis of PAIS includes many diseases such as gonadal dysgenesis, 17α-hydroxylase and 17-beta-hydroxysteroid dehydrogenase deficiency, and 5-alfa-reductase (5α-RD2) deficiency [1]. Adult women with CAIS and intact gonads have T levels either within or above the normal range for men and boys and luteinizing hormone (LH) concentrations inappropriately increased, thus resembling a hormone-resistant state. Follicle-stimulating hormone (FSH) and inhibin are generally normal. Estrogen levels may be increased compared to male reference values, mainly due to the peripheral aromatization of testosterone, although they remain below the average level of women of reproductive age. The profile of LH and T concentrations is less suggestive of hormone resistance when complete androgen insensitivity syndrome presents in infancy. Serum AMH is usually above the male range. Hormonal profile in children is performed with a stimulation test with human chorionic gonadotropin (hCG) and the measurement of androstenedione, T, and DHT in serum 72 h later [10]. 

Recently, the role of the LC-MS/MS hormonal profile has been suggested in the differential diagnosis between AIS and 5α-RD2. Indeed, DHT and T were both significantly lower in patients with 5a-RD2 than AIS. The T/DHT ratio was higher in 5a-RD2 than in AIS or healthy men. 11ß-hydroxyandrostenedione (11OHA4) and 11-ketoandrostenedione (11KA4) were also lower in patients with 5α-RD2 than those in patients with AIS. In contrast, 11β-hydroxytestosterone (11OHT) was higher in 5α-RD2 than AIS [11].

In our patient, the hormonal profile at the newborn screening for 21-hydroxylase deficiency was unremarkable. The DHT levels and testosterone/DHT ratio excluded 5-a reductase deficiency while the AMH levels were compatible with the male genotype. The chromosome analysis revealed a 46XY genotype, thus ruling out gonadal dysgenesis. The absence of internal genitalia at the abdominal ultrasound confirmed the suspicion of CAIS. Therefore, the molecular analysis of the AR gene was then performed resulting in a mutation in exon 4 of the AR gene.

At present, more than 1000 mutation sites of the AR gene are known, including substitution, translocation, deletion, and insertion, located at various loci of the AR gene [10]. 

This is the first report of a novel mutation in exon 4 of the AR gene. The mutation is a single-nucleotide substitution (C to A) at position 2108 (c.2108 C>A), resulting in the substitution of a serine with a tyrosine at codon 703 onto codified protein (p.Ser703Tyr).

About 100 mutations in exon 4 of the AR gene are known to be responsible for CAIS, PAIS, or MAIS (https://androgendb.mcgill.ca/ accessed on 31 May 2023). Two previous studies had described the mutations of the gene in the loci adjacent to that reported in our case. Pinsky et al. described a mutation in the same codon due to a single-nucleotide substitution (T to G) at position 2107 (c.2107 T>G), resulting in the substitution of a serine with an alanine (p.Ser703Ala). In this case, the patient showed a CAIS phenotype with normal female genitalia [12].

Radnayr et al., however, described a patient with PAIS, with ambiguous genitalia, who presented a mutation in codon 704, caused by the substitution of a serine with a glycine (p.Ser704Gly) due to a single-nucleotide substitution (A>G) at position 2110 (c.2110A>G) [13].

The AR gene is organized into eight exons and seven introns and encodes the androgen receptor protein, a member of the nuclear receptor superfamily, designated NR3C4 (nuclear receptor subfamily 3, group C, member 4). The AR protein consists of a 920 amino acid sequence and has a molecular mass of 110 kDa. The AR is a single-stranded polypeptide with four major structural domains [14]. The N-terminal domain (NTD) includes the transactivator domain, which activates and regulates the transcription of target genes and contributes to the final three-dimensional structure of the receptor [15]. 

The DNA-binding domain (DBD) is formed by many cysteine residues, resulting in a tertiary structure called a “zinc finger”, which is particularly suitable for binding with hormone response elements (HREs). The ligand-binding domain (LBD) includes androgen-specific binding sites, different coactivation transcription factors, and the activation function-2 (AF-2) region. It promotes receptor interaction with heat shock proteins (HSPs) in the cytoplasm and then with androgenic hormone, leading to AR migration into the nucleus [2,15,16,17]. A characteristic feature of the AR is the N-terminal/C-terminal interaction between the AF-1 (N-terminal) and AF-2 (C-terminal) subdomains, aimed at stabilizing the binding between the receptor and its ligand and slowing its dissociation.

According to a VarSite computational analysis, the residue at sequence position 703 in AR is a serine which has a neutral side chain. On the other hand, the identified missense variant causes a substitution of Ser703 with a tyrosine which has an aromatic side chain which can stack against each other. The variant CADD score is 24.90 resulting in a possibly deleterious effect. From the literature, variants in Ser703 are already known to be related to AIS [17]. The Ser703 is highly conserved (conservation = 0.8 from 172 aligned protein seqs) showing an important role in LBD for the function of the protein. 

Based on clinical evidence and molecular and computational data, we hypothesize that variant S703Y in the AR gene may have a pathogenetic role related to AIS. 

Besides describing a new variant in the AR gene, we hereby emphasize the importance of a multidisciplinary approach that allows a very early diagnosis of AIS. 

In conclusion, the discovery of a new variant in the AR gene is crucial for several reasons, especially in the context of androgen insensitivity syndrome (AIS): (1) improved diagnosis: the identification of novel variants in the AR gene helps to confirm the diagnosis of AIS. (2) Understanding phenotypes: The different variants of the AR gene can give rise to a spectrum of phenotypes, from complete androgen insensitivity syndrome (CAIS) to partial (PAIS) and mild (MAIS). The knowledge of these variants helps predict the severity of the condition and personalize patient management. (3) Personalized treatment: understanding these variants will help predict the condition’s severity and personalize patient management. (4) Genetic counseling: the discovery of new variants provides information about the condition. (5) Research and development: each new variant contributes to the scientific understanding of AIS, particularly in the case of AR. 

However, one limitation of our study is the lack of functional studies meant to confirm our hypothesis, currently based on clinical data and in silico models. It would be worth conducting functional studies aimed at validating our hypothesis on the pathogenetic role of the newly identified variant.

## Figures and Tables

**Table 1:** Serum androgens, AMH, and 17α-Hydroxy-Progesterone at the time of presentation (compared with the recommended pediatric reference ranges).

Laboratory Test	Result	Normal Range
AMH (ng/mL)	45.9	/
Testosterone (ng/mL)	0.73	<0.025
Dihydrotestosterone (pg/mL)	438.6	24–368
d-4-androstenedione (ng/mL)	0.8	0.3–6.5
17α-Hydroxy-Progesterone (ng/mL)	1.7	<20

**Table 2:** Custom NGS panel of 50 genes involved in DSD.

GENE	OMIM	REFSEQ	GENE	OMIM	REFSEQ
*AKR1C2*	600450	NM_001354.5	*LEP*	164160	NM_000230.2
*AKR1C4*	600451	NM_001818.3	*LHCGR*	152790	NM_000233.3
*ANOS1*	300836	NM_000216.3	*MAMLD1*	300120	NM_005491.3
*AR*	313700	NM_000044.3	*NR0B1*	300473	NM_000475.4
*ATRX*	300032	NM_000489.3	*NR3C1*	138040	NM_001018077.1
*BMP15*	300247	NM_005448.2	*NR5A1*	184757	NM_004959.4
*CHD7*	608892	NM_017780.3	*POR*	124015	NM_000941.2
*CYB5A*	613218	NM_001914.3	*PROK2*	607002	NM_00112128.1
*CYP17A1*	609300	NM_000102.3	*PROKR2*	607623	NM_144773.3
*CYP11B1*	610613	NM_000497.3	*PROP1*	601538	NM_006261.4
*CYP19A1*	107910	NM_000103.3	*RSPO1*	609595	NM_001038633.3
*DHH*	605423	NM_021044	*RXFP2*	606655	NM_130806.3
*DMRT1*	601898	NM_004122.2	*SOX9*	608160	NM_000346.3
*DMRT2*	602424	NM_021951.2	*SRD5A2*	607306	NM_000348.3
*FGF8*	600483	NM_006119.4	*STAR*	600612	NM_000349.2
*FGFR1*	136350	NM_023110.2	*TAC3*	162330	NM_001178054.1
*FGFR2*	176943	NM_000141.4	*WDR11*	606417	NM_018117.11
*FSHB*	136530	NM_000510.2	*WT1*	607102	NM_024426.4
*GATA4*	600576	NM_002052.3	*ZFPM2*	603693	NM_012082.3
*GNRH1*	152760	NM_001083111.1	*HSD17B3*	605573	NM_000197.1
*GNRHR*	138850	NM_000406.2	*HSD17B4*	601860	NM_000414.3
*HESX1*	601802	NM_003865.2	*HSD3B2*	613890	NM_000198.3
*AMHR2*	600956	NM_020547.3	*INSL3*	146738	NM_005543.4
*CYP11A1*	118485	NM_000781.3	*MAP3K1*	600982	NM_005921.2
*FSHR*	136435	NM_000145.3	*SRY*	480000	NM_003140.3

## Data Availability

The data presented in this study are available on request from the corresponding author due to privacy.

## Supplementary Materials

The following supporting information can be downloaded at: https://www.mdpi.com/article/10.3390/biomedicines12081742/s1, Table S1: Timetable of gradient elution for detection of CORT, 21-DC, 11-DC, A4, and 17-OHP; Table S2: Multiple Reaction Monitoring (MRM) functions and settings for detection of cortisol, 21-deoxycortisol, 11-deoxycortisol, 4-androstene-3,17-dione, and 17α-hydroxyprogesterone; Table S3: Linearity values of the LC-MS/MS method after analyzing batches over 3 non-consecutive days; Table S4: Precision and accuracy values of the LC-MS/MS method after analyzing batches over 3 non-consecutive days.
