# Supplementary material for: A Very Early Diagnosis of Complete Androgen Insensitivity Syndrome Due to a Novel Variant in the AR Gene: A Neonatal Case Study
Source: Biomedicines. 2024 Aug 2;12(8):1742. doi: 10.3390/biomedicines12081742 (PMC11351560; doi:10.3390/biomedicines12081742)
Supplement: Supplementary file 1 [file biomedicines-12-01742-s001.zip › biomedicines-3118046-supplementary.pdf]

biomedicines

MDPI

Case Report

A Very Early Diagnosis of Complete Androgen Insensitivity Syndrome Due to a Novel Variant in the AR gene

Rossella Ferrante[1], Stefano Tumini[2], Maria Alessandra Saltarelli[3], Sara Di Rado[1], Vincenzo Scorrano[1], Maria Lucia Tommolini[1,4], Mirco Zucchelli[1,4], Federico Lauriola[3], Gabriele Lisi[5,6], Giuseppe Lauriti[5,6], Nino Marino[5], Liborio Stuppia[1,7], Claudia Rossi[1,4*], and Ines Bucci[1,6]

[1] Center for Advanced Studies and Technology (CAST), "G. d'Annunzio" University of Chieti-Pescara, 66100 Chieti, Italy; rossella.ferrante@unich.it (R.F.); sara.dirado@phd.unich.it (S.D.R.); vincenzo.scorrano@unich.it (V.S.); maria.tommolini@unich.it (M.L.T.); m.zucchelli@unich.it (M.Z.); liborio.stuppia@unich.it (L.S.); ines.bucci@unich.it (I.B.)
[2] Department of Maternal and Child Health, UOSD Regional Center of Pediatric Diabetology, Chieti Hospital, 66100 Chieti, Italy; stefano.tumini@asl2abruzzo.it
[3] Department of Pediatrics, University of Chieti, 66100 Chieti, Italy; mariaalessandra.saltarelli@gmail.com (M.A.S.); federicolauriola@hotmail.it (F.L.)
[4] Department of Innovative Technologies in Medicine and Dentistry, "G. d'Annunzio" University of Chieti-Pescara, 66100 Chieti, Italy
[5] Pediatric Surgery Unit, Maternal and Child Health Department, Pescara Public Hospital, 65121 Pescara, Italy; gabriele.lisi@unich.it (G.L.); giuseppe.lauriti@unich.it (G.L.); nino.marino@asl.pe.it (N.M.)
[6] Department of Medicine and Aging Science, "G. d'Annunzio" University of Chieti-Pescara, 66100 Chieti, Italy
[7] Department of Psychological, Health and Territory Sciences, "G. d'Annunzio" University of Chieti-Pescara, 66100 Chieti, Italy
* Correspondence: claudia.rossi@unich.it; Tel.: +39-0871-541290

Citation: Ferrante, R.; Tumini, S.; Saltarelli, M.A.; Di Rado, S.; Scorrano, V.; Tommolini, M.L.; Zucchelli, M.; Lauriola, F.; Lisi, G.; Lauriti, G.; et al. A Very Early Diagnosis of Complete Androgen Insensitivity Syndrome Due to a Novel Variant in the AR Gene: A Neonatal Case Study. *Biomedicines* **2024**, *12*, 1742. https://doi.org/10.3390/biomedicines12081742

Academic Editor(s): Felipe Javier Chaves-Martinez

Received: 6 July 2024
Revised: 29 July 2024
Accepted: 31 July 2024
Published: 2 August 2024

Copyright: © 2024 by the authors. Licensee MDPI, Basel, Switzerland. This article is an open access article distributed under the terms and conditions of the Creative Commons Attribution (CC BY) license (https://creativecommons.org/licenses/by/4.0/).

Routinely Newborn Screening Analysis for Congenital Adrenal Hyperplasia

Dried blood spot (DBS) samples for NBS are punched out into 3.2 mm-disks to perform high-throughput analyses for the detection of over than 50 IEMs. More precisely, five 3.2 mm DBS disks are used to test by immunofluorimetric assays congenital hypothyroidism (CH), cystic fibrosis (CF), galactosemia, biotinidase deficiency and congenital adrenal hyperplasia (CAH), respectively. Other two DBS disks are employed for flow injection-tandem mass spectrometry analyses (FIA-MS/MS), the first one for aminoacidemias, urea cycle disorders, organic acidemias [4], fatty acid oxidation disorders [5], and immunodeficiencies [6], the second one for lysosomal storage diseases [7]. The eighth DBS disk is used to screen newborns for Spinal Muscular Atrophy (SMA) by real-time PCR analysis [8].

In particular, NBS for CAH is based on the quantitative determination of human 17 α -OH-progesterone (17-OHP) in blood specimens dried on filter paper using the GSP® instrument and the GSP Neonatal 17-OHP kit, an in vitro diagnostic kit (PerkinElmer, Turku, Finland). The assay consists of a solid phase, time-resolved fluoroimmunoassay based on the competitive reaction between europium-labeled 17-OHP and sample 17-OHP for a limited amount of binding sites on 17-OHP specific polyclonal antibodies.

Materials and DBS samples preparation for steroid profiling by UPLC-MS/MS analysis

DBS sample preparation for cortisol, 21-deoxycortisol, 11-deoxycortisol 4-androstene-3,17-dione, 17α-hydroxyprogesterone

A working solution containing 2.5 ng ISs, with 4-androstene-3,17-dione (A4) D7, cortisol (CORT) D3, 17α-hydroxyprogesterone (17-OHP) D8, 11-deoxycortisol (11-DC) D2, and 21-deoxycortisol (21-DC) D8 purchased from Spectra 2000, was prepared by diluting the IS stock solutions with 95:5 (v/v) methanol/water. Calibrators and Quality

Controls (QCs) were purchased from LabSystem Diagnostics (Helsinki, Finland). 250 µL of working solution were added to two 3.2 mm diameter punches from DBS samples, calibrators, and QCs. After been incubated at T°C room for 30 minutes at 700 rpm, supernatants were transferred in other tubes and evaporated till dryness. The residues were reconstituted with 100 µL of 60:40 (v/v) water 0.05 mM ammonium fluoride (NH_4) / methanol and finally transferred into vials for LC-MS/MS analysis. The sample volume injected into the ion source was 20 µL.

LC-MS/MS conditions

The LC-MS/MS system consisted of an ACQUITY™ UPLC™ I-Class system comprised of a Binary Solvent Manager (BSM) and a Sample Manager with Flow-Through Needle (SM-FTN) coupled to a Xevo® TQ-S micro mass spectrometer (Waters Corporation, Milford, MA, USA). The system operated in positive electrospray ionization (ESI^+). The run time was 2.50 minutes, injection-to-injection. For chromatographic separation was employed a CORTECS C18 2.7µM, 50mm x 2.1mm (Waters co.), using a CORTECS C18 2.7µM VanGuard pre-culumn; column thermostated at 50 °C and the autosampler at 10 °C. Mobile phase (solvents purchased from Carlo Erba Reagents) was composed of water (A) 0.05 mM ammonium fluoride (NH_4) and methanol (B). Flow rate was 1.0 mL/min. LC gradient is elucidated in Table S1. Parameters referring to MRM functions for the detection of CORT, 21-DC, 11-DC, A4, 17-OHP are reported in Table S2. Data were processed using TargetLynx™ XS software (Waters Corporation, Milford, MA, USA). Linearity, precision, and accuracy values were evaluated and are summarized in Supplementary Tables S3 and S4 (ST3 and ST4).

Table S1. Timetable of gradient elution for detection of CORT, 21-DC, 11-DC, A4, 17-OHP.

N.	Time (min)	Flow Rate (mL/min)	%A	%B	Curve
1	Initial	1.000	60.0	40.0	Initial
2	0.10	1.000	60.0	40.0	6
3	0.25	1.000	55.0	45.0	6
4	0.75	1.000	55.0	45.0	6
5	0.90	1.000	37.5	62.5	6
6	1.15	1.000	5.0	95.0	11
7	1.40	1.000	60	40	11

Table S2. Multiple Reaction Monitoring (MRM) functions and settings for detection of cortisol, 21-deoxycortisol, 11-deoxycortisol, 4-androstene-3,17-dione, 17α-hydroxyprogesterone.

MRM Function	Time Window (min)	Analyte	Dwell time (s)	Transitions (m/z)	Cone (V)	Collision Energy (CE)
1	0-2.50	A4 (Quant.)	0.010	287.30 > 97.10	50.0	20.0
2	0-2.50	A4 (Qual.)	0.010	287.30 > 109.10	50.0	20.0
3	0-2.50	A4 D7 IS	0.010	294.30 > 100.00	50.0	20.0
4	0-2.50	17-OHP (Quant.)	0.010	331.30 > 97.10	50.0	20.0
5	0-2.50	17 -OHP (Qual.)	0.010	331.30 > 109.10	50.0	20.0
6	0-2.50	17-OHP D8 IS	0.010	339.30 > 100.10	50.0	20.0

7	0-2.50	11-DC (Quant.)	0.010	347.30 > 97.10	50.0	22.0
8	0-2.50	11-DC (Qual.)	0.010	347.30 > 109.10	50.0	22.0
9	0-2.50	11-DC D2 IS	0.010	349.30 > 97.00	50.0	22.0
10	0-2.50	21-DC (Quant.)	0.010	347.30 > 311.10	50.0	14.0
11	0-2.50	21-DC (Qual.)	0.010	347.30 > 121.20	50.0	22.0
12	0-2.50	21-DC D8 IS	0.010	355.30 > 319.30	50.0	14.0
13	0-2.50	CORT (Quant.)	0.010	363.30 > 121.10	50.0	22.0
14	0-2.50	CORT (Qual.)	0.010	363.30 > 91.10	50.0	50.0
15	0-2.50	CORT D3 IS	0.010	366.30 > 121.00	50.0	22.0

Table S3. Linearity values of the LC-MS/MS method after analyzing batches over 3 non-consecutive days. R2 values were found to be always greater than 0.99 for 4A, 17-OHP, 11-DC, 21-DC, CORT.

Compound	Concentration Range (ng/mL)	R2
**4A**	1.32 - 298.580	0.99
**17-OHP**	1.30 - 283.660	0.99
**11-DC**	1.46 - 340.23	0.99
**21-DC**	1.27 - 285.93	0.99
**CORT**	1.66 - 383.84	0.99

Table S4. Precision and accuracy values of the LC-MS/MS method after analyzing batches over 3 non-consecutive days. N.C.: nominal concentration; SD: standard deviation; %CV: coefficient of variation; %RE: relative error.

**4A**	**N.C.**	**1,32**	**2,77**	**5,32**	**10,7**	**76,65**	**154,48**	**298,58**
	**Average**	1,34	2,72	5,46	10,51	74,92	151,18	308,93
	**SD**	0,021	0,064	0,26	0,39	3,48	8,96	28,11
	**%CV**	1,57	2,35	4,76	3,71	4,64	5,93	9,1
	**%RE**	1,51	-1,81	2,63	-1,78	-2,26	-2,14	3,47
**17-OHP**	**N.C.**	1,3	2,62	5,1	10,09	73,02	148,93	283,66
	**Average**	1,29	2,52	5,2	10,21	72,84	143,68	298,46
	**SD**	0,075	0,11	0,33	0,76	3,25	8,21	27,34
	**%CV**	5,81	4,37	6,35	7,44	4,46	5,71	9,16
	**%RE**	-0,77	-3,82	1,96	1,19	-0,25	-3,53	5,22
**11-DC**	**N.C.**	1,46	3,1	6,04	12,22	86,01	172,99	340,23
	**Average**	1,46	3,05	6,23	12,41	86,14	168,04	340,89
	**SD**	0,021	0,032	0,39	0,48	3,15	8,54	32,17
	**%CV**	1,44	1,05	6,26	3,87	3,66	5,08	9,44
	**%RE**	0	-1,61	3,15	1,55	0,15	-2,86	0,19
**21-DC**	**N.C.**	1,27	2,64	5,14	10,27	72,39	148,39	285,93
	**Average**	1,31	2,47	5,10	10,44	73,17	143,99	298,62

	**SD**	0,062	0,25	0,27	0,43	2,34	5,74	30,33
	**%CV**	4,73	10,12	5,29	4,12	3,2	3,99	10,16
	**%RE**	3,15	-6,44	-0,78	1,66	1,08	-2,97	4,44
**CORT**	**N.C.**	1,66	3,65	7	14,1	98,95	201,04	383,84
	**Average**	1,68	3,46	7,28	14,10	98,52	193,26	399,16
	**SD**	0,07	0,25	0,55	0,67	3,84	5,15	39,77
	**%CV**	4,17	7,23	7,55	4,75	3,9	2,66	9,96
	**%RE**	1,2	-5,21	4	0	-0,43	-3,87	3,99

References

4. Rossi, C.; Cicalini, I.; Rizzo, C.; Zucchelli, M.; Consalvo, A.; Valentinuzzi, S.; Semeraro, D.; Gasparroni, G.; Brindisino, P.; Gazzolo, D.; Dionisi-Vici, C.; De Laurenzi, V.; Pieragostino, D. A False-Positive Case of Methylmalonic Aciduria by Tandem Mass Spectrometry Newborn Screening Dependent on Maternal Malnutrition in Pregnancy. *Int J Environ Res Public Health* **2020**, *17(10):3601,*

5. Janeiro, P.; Jotta, R., Ramos, R., Florindo, C., Ventura, F. V., Vilarinho, L., Tavares de Almeida, I., & Gaspar, A. Follow-up of fatty acid β-oxidation disorders in expanded newborn screening era. *European journal of pediatrics* **2019**, 178(3):387-394.

6. Malvagia, S.; Funghini, S., Della Bona, M., Ombrone, D., Mura, M., Damiano, R., Ricci, S., Cortimiglia, M., Azzari, C., & la Marca, G. The successful inclusion of ADA SCID in Tuscany expanded newborn screening program. *Clinical chemistry and laboratory medicine* **2021**, *59*(10), *59(10):e401-e404.*

7. Rossi, C.; Ferrante, R., Valentinuzzi, S., Zucchelli, M., Buccolini, C., Di Rado, S., Trotta, D., Stuppia, L., Federici, L., & Aricò, M. Noninvasive DBS-Based Approaches to Assist Clinical Diagnosis and Treatment Monitoring of Gaucher Disease. *Biomedicines* **2023**, *11(10):2672*.

8. Angilletta, I.; Ferrante, R., Giansante, R., Lombardi, L., Babore, A., Dell'Elice, A., Alessandrelli, E., Notarangelo, S., Ranaudo, M., Palmarini, C., De Laurenzi, V., Stuppia, L., & Rossi, C. Spinal Muscular Atrophy: An Evolving Scenario through New Perspectives in Diagnosis and Advances in Therapies. *International journal of molecular sciences* **2023**, *24(19):14873*.

Disclaimer/Publisher's Note: The statements, opinions and data contained in all publications are solely those of the individual author(s) and contributor(s) and not of MDPI and/or the editor(s). MDPI and/or the editor(s) disclaim responsibility for any injury to people or property resulting from any ideas, methods, instructions or products referred to in the content.
